# Alignment and clustering of phylogenetic markers - implications for microbial diversity studies

**DOI:** 10.1186/1471-2105-11-152

**Published:** 2010-03-24

**Authors:** James R White, Saket Navlakha, Niranjan Nagarajan, Mohammad-Reza Ghodsi, Carl Kingsford, Mihai Pop

**Affiliations:** 1 Applied Mathematics and Scientific Computation Program, University of Maryland - College Park, College Park, MD, 20742, USA; 2 Department of Computer Science, University of Maryland - College Park, College Park, MD, 20742, USA; 3 Center for Bioinformatics and Computational Biology, University of Maryland - College Park, College Park, MD, 20742, USA; 4 Computational and Mathematical Biology Program, Genome Institute of Singapore, 138672, Singapore

## Abstract

**Background:**

Molecular studies of microbial diversity have provided many insights into the bacterial communities inhabiting the human body and the environment. A common first step in such studies is a survey of conserved marker genes (primarily 16S rRNA) to characterize the taxonomic composition and diversity of these communities. To date, however, there exists significant variability in analysis methods employed in these studies.

**Results:**

Here we provide a critical assessment of current analysis methodologies that cluster sequences into operational taxonomic units (OTUs) and demonstrate that small changes in algorithm parameters can lead to significantly varying results. Our analysis provides strong evidence that the species-level diversity estimates produced using common OTU methodologies are inflated due to overly stringent parameter choices. We further describe an example of how semi-supervised clustering can produce OTUs that are more robust to changes in algorithm parameters.

**Conclusions:**

Our results highlight the need for systematic and open evaluation of data analysis methodologies, especially as targeted 16S rRNA diversity studies are increasingly relying on high-throughput sequencing technologies. All data and results from our study are available through the JGI FAMeS website http://fames.jgi-psf.org/.

## Background

Microbial diversity within the human body has recently been quantified through 16S rRNA surveys [1-4] and metagenomic methods. The latter provide a detailed view of the genomic composition and functional potential of human-associated microbial communities through shotgun sequencing [5]. However this level of resolution comes with a high price-tag - billions of base-pairs need to be sequenced to ensure a sufficient level of sampling of complex communities [6] such as those found in the human gastrointestinal tract. 16S rRNA surveys provide limited insight into the composition of the commensal microbiome. However, due to substantially lower costs, such studies are currently the only practical approach for studying large numbers of samples (such as those generated in a clinical setting). In this paper, we explore the limitations of unsupervised clustering methods used to analyze 16S rRNA data, particularly the large impact of small changes in the parameters of the analysis process. We specifically focus on the most common strategy - the clustering of 16S rRNA sequences into a collection of *operational taxonomic units *(OTUs) or *phylotypes *on the basis of sequence similarity. An evaluation of taxonomic classification through database searches [4] or other fully supervised classification methods [7] are beyond the scope of this paper. Fully supervised approaches are inherently limited due to the current undersampling of the global microbial population, only allowing accurate classification of a fraction of sequences (as low as 20% in some studies [1]). As an alternative, the unsupervised clustering of 16S sequences allows for detection of species-like units in complex unknown bacterial environments, even if a precise taxonomic identity cannot be assigned to these units.

The OTU clustering process typically begins by constructing a multiple alignment (MSA) of the 16S rRNA sequences. The MSA is then used to estimate pairwise distances between individual sequences, expressed as the fraction of nucleotides that have changed as the sequences have evolved from their most recent common ancestor. To accurately reflect evolutionary processes, the distances inferred from the MSA are corrected using one of several models of evolution [8]. The distances are provided as input to a hierarchical clustering algorithm (nearest neighbor, furthest neighbor, or average neighbor/UPGMA are commonly used). Sub-clusters or OTUs are defined by applying a distance threshold, selected to roughly approximate a specific taxonomic level: thresholds between 1-3% are typically used to approximate individual species, 5% for individual genera, 15% for classes, etc. [9-11]. Note that alternative approaches to OTU creation exist (e.g. those that avoid MSAs (CD-hit) [12], or use databases [13]), but the general technique we describe above has been widely employed across bacterial diversity studies.

The choice of MSA, distance correction, clustering algorithm, and distance threshold vary considerably between studies, and to our knowledge, there has been no rigorous evaluation of the impact of methodological choices on the ecological conclusions of the analysis process. Previously, simulated datasets have been successfully used to evaluate methods for the assembly and binning of metagenomic data [14]. In this study, we rely on simulated datasets to provide a comprehensive assessment of the extent to which individual parameters in the OTU clustering process affect the estimated diversity and composition of a microbial environment. We evaluate methodological choices in terms of how well the clustering of sequences into a set of OTUs matches the clustering imposed by the known (i.e. database annotated) membership of the sequences to individual bacterial species. The OTU clusterings are compared using a mathematically robust metric - the Variation of Information (VI) [15] - an information-theoretic measure of the amount information lost or gained by changing from one clustering to another.

Through this evaluation framework, we will demonstrate that OTUs are highly sensitive to small changes in the clustering methodology and reveal a surprising observation that reducing the stringency of clustering distance thresholds tends to produce more accurate species-level representations of a community. We will further assess the impact of OTU variability on common ecological measures of diversity and provide an example of how semi-supervised clustering could produce more robust OTU structures by accounting for varying evolutionary rates across the microbial phylogeny.

## Methods

### Creation of simulated datasets

Our simulated dataset comprises 1677 16S rRNA sequences from the RDP database (release 9.57) [16], that satisfy the following properties: (i) at least 800 bp long; (ii) can be aligned by NAST [17] requiring a match to the template alignment of at least 75% identity; (iii) have full taxonomic identification at all levels from phylum to species according to the RDP taxonomy (Garrity et al. [18]); and (iv) the taxonomic identity of each of the sequences is confirmed by the RDP Naïve Bayesian classifier [7] at ≥ 95% confidence, GreenGenes SimRank [19], and through BLASTN [20] searches against a reduced RDP database (after filtering out the set of simulated sequences). Thus, while it is impossible to verify the correct taxonomic membership of all these sequences, we can guarantee that their annotation is consistent across multiple databases and classification procedures. These sequences were largely obtained from bacterial isolates (96.2%) and had unambiguous taxonomic assignment at the species level. Our simulated environment spans 49 species, 46 genera, 37 families, 21 orders, 12 classes, and seven phyla including Proteobacteria, Bacteroidetes, Firmicutes, and Actinobacteria. Alpha-, Beta-, and Gammaproteobacteria make up 66% of the sequences in roughly equal proportions. A similar class distribution has been reported for microbial communities found in the phyllosphere of the Atlantic rainforest [21].

It is important to note that, by choice, these sequences are high quality and belong to relatively well-characterized taxonomic groups. Therefore any results obtained on this highly curated dataset represent an upper bound on the performance that can be achieved when analyzing noisy data from environmental surveys.

### Parameter evaluation: multiple sequence alignments, distance corrections, clustering methods and distance thresholds

All 1677 sequences were aligned using MUSCLE [22], ClustalW [23], and NAST [17] using default parameters. ClustalW was run with the "Fast" option for pairwise alignments, a heuristic setting that dramatically improves running time (scaling roughly linearly as the number of sequences increases, as opposed to cubic running times necessary for the full alignment procedure) at the cost of a lower quality alignment. In the NAST alignment, all columns containing only gaps were removed, and each MSA was trimmed so that every sequence spanned the entire alignment. The trimmed MSAs covered the range of V2, V3, and V4 hypervariable regions within the *E. coli *O157:H7 str. TW14359 16S rRNA gene.

Distance matrices were constructed with DNADIST from the PHYLIP package [8] using each of the Jukes-Cantor, Kimura 2-parameter, and Felsenstein84 corrections, keeping the remaining parameters at their default values (in particular, insertions/deletions are ignored in the distance computation). (Olsen and F84 distance-corrected matrices were also generated using the ARB package [24] in a later analysis not included in our combinatorial search.) Distance matrices were then provided as input to DOTUR [25], and the resulting data clustered using, in turn, nearest neighbor, average neighbor, and furthest neighbor clustering procedures on each individual matrix. Individual clusters were generated, for each clustering algorithm, by varying a distance threshold parameter ranging from 0.00 to 0.45 (incremented by 0.01). The distance threshold D has a different meaning depending on the particular algorithm: in furthest/average/nearest neighbor, two clusters are merged if the maximum/average/minimum distance between any two elements in the combined cluster is less than or equal to D.

The process described above generated 1242 sets of clusters/OTUs (3 MSAs × 3 distance corrections × 3 clustering algorithms × 46 distance thresholds), 749 of which are distinct (i.e. multiple parameter combinations lead to the same set of OTUs).

### Comparing clusterings

We employed the Variation of Information (VI) metric [15] as a measure of similarity between two partitions (or clusterings) of a given set [15]. For this study, the set comprises the 1677 16S sequences selected for the artificial environmental sample. Mathematically, a given clustering *C*, is a partition of a set *S *into disjoint subsets (clusters) where:

If there are *m *elements in set *S*, and we let *m*_*i *_be the number of elements in cluster *C*_*i*_, then to compute the Variation of Information between two clusterings, we first find the probability that a randomly selected sequence is in a particular cluster, that is, . Given this discrete probability distribution, the uncertainty of the random variable *i *is the entropy associated with clustering *C*, defined as:

Now, suppose we have two clusterings *C *= {*C*_1_, *C*_2_, ..., *C*_*M*_}, and *D *= {*D*_1_, *D*_2_, ..., *D*_*M*'_}. Then we calculate the joint distribution  describing the similarity of all pairs of clusters between *C *and *D*. The *mutual information *between the clusterings *C *and *D *is then defined to be , and finally, the variation of information between *C *and *D *is defined as the sum of the individual clustering entropies less 2 times the mutual information:

If *C *and *D *are identical clusterings, then *H(C) = H(D) = I(C,D)*, and the *VI *= 0. The VI distance is a true metric, satisfying symmetry, non-negativity, and the triangle inequality.

In order to provide a reference set of VI distances for known clusters, we measured the VI between the "true" species clustering (i.e. annotated according to the RDP taxonomy) and the annotated phylum, class, order, family, and genus clusterings (Additional File 1: Table S1).

### VI-cut method for defining OTUs

VI-cut is a procedure that finds a clustering from a hierarchical tree decomposition *T *that optimally matches a subset of known labels, as defined by the Variation of Information metric [26]. A clustering is defined by choosing a set of nodes in *T*. Each chosen node *c *corresponds to a single cluster consisting of all the leaves (*i.e*. sequences) in the subtree rooted at *c*. Collectively, the chosen nodes correspond to a node-cut *K*, which induces a non-overlapping clustering *A*_*K*_. Let *D *represent the partial clustering of labeled sequences such that sequences with the same label are grouped together. The VI-cut algorithm finds the *A*_*K *_that minimizes the VI distance to *D*:

Although there are exponentially many possible node-cuts in *T*, an optimal node-cut can be found efficiently using dynamic programming [26]. As described, the VI-cut algorithm creates overly-general clusters when faced with sparsely labeled data. To correct for this, we used a strategy called *forbidden nodes*. Specifically, any node *n *with a corresponding diameter ≥ 0.07 was labeled as "forbidden", and we required that the clusters produced by VI-cut do not contain any forbidden nodes. This requirement implicitly restricts the maximum diameter of an OTU to 7% divergence. To incorporate forbidden nodes into the VI-cut algorithm, we first ran the standard VI-cut algorithm. Any cluster that contained a forbidden node *n *was then sub-divided into non-overlapping clusters by identifying subtrees dominated by *n *that do not contain any forbidden nodes.

### Assessment of OTU methodology factors using ANOVA

To isolate the individual impact of each component in an OTU methodology, the 200 methodologies resulting in the lowest VI distance from the annotated species clustering were analyzed using multi-way analysis of variance (ANOVA) considering four factors: multiple sequence alignment, evolutionary distance correction, clustering algorithm, and distance threshold.

## Results

### OTU variability

**Figure 1:**
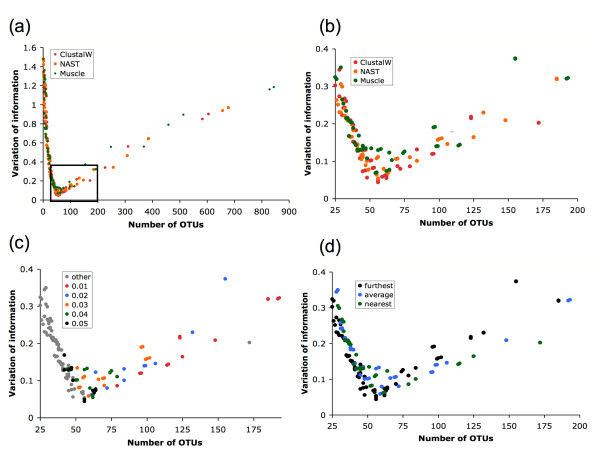
**OTU variability observed through comprehensive methodology search**. (a) The number of OTUs found versus the VI distance from the annotated species clustering for 749 OTU sets. Generally, smaller clustering distances lead to many OTUs while larger clustering distances result in very few OTUs, both of which poorly approximate the species-level structure in the sample. Near 49 OTUs, the true number of species in the simulated sample, the OTU sets are relatively closer to the true species structure. Detail of the lower-left corner of (a) re-colored by (b) MSA, (c) distance threshold, and (d) clustering algorithm.

The results of our analysis (summarized in Figure 1) reveal a large variation in the level of concordance of the OTU clustering with the annotated species-level composition of the environment when varying methodology parameters. We specifically highlight the parameters with most impact - distance threshold (Figure 1C), MSA (Figure 1A and 1B), and clustering strategy (Figure 1D) - parameters that accounted for 56%, 33%, and 7% of the total variation, respectively (confirmed by ANOVA, see Table 1). Distance correction measures did not significantly contribute to the variability (F-test, *F *= 0.002, *P *= 0.998), implying that such corrections are roughly equivalent within the short evolutionary time frame defining a microbial species. We extended this comparison to include the Olsen distance correction in ARB [24], which we found produced OTUs virtually identical to those created using the F84 correction.

**Table 1:** Multi-way ANOVA table assessing components used in OTU methodologies.

Parameter	Sum of Squares	Degrees of freedom	**Mean Sq**.	*F*	Prob >*F*
Distance threshold	0.4411	11	0.0401	23.0160	< 0.0001
MSA	0.0480	2	0.0240	13.7843	< 0.0001
Clustering	0.0099	2	0.0050	2.8503	0.0604
Distance correction	< 0.0001	2	< 0.0001	0.0020	0.9980
Error	0.3171	182	0.0017		
Total	0.7910	199	0.0708		

The factor with the largest effect on the quality of the OTUs was the distance threshold, followed by the MSA, and then the clustering algorithm. The distance correction explained < 0.01% of the variance and no statistically significant difference could be detected between the corrections (P = 0.998).

None of the parameter combinations perfectly captured the annotated species composition (49 OTUs, VI distance = 0). Large variation in the OTU content is observed even when we fix the similarity threshold to 0.01 (approximately strain-level) - the number of OTUs ranges from 79 to 248 at this similarity level, depending on the choice of MSA or clustering strategy. Surprisingly, the OTU clustering closest to the annotated species clustering was obtained using a similarity threshold of 0.05 - a value larger than the cutoffs usually used to approximate the species-level composition of an environment (0.01-0.03 [1,3,11]). In terms of alignment, methodologies employing ClustalW or NAST were roughly similar and performed slightly better than those using MUSCLE (Figure 1B). However, applying our validation methodology to ten randomly selected subsets of the original data, we discovered that no MSA program consistently outperformed the others (see *Consistency of methods across multiple datasets *below). In terms of clustering strategy, furthest neighbor resulted in the best agreement with the annotated species structure of our environment (Figure 1D).

Even the combination of analysis parameters with the lowest VI distance (ClustalW, furthest neighbor, 0.05 distance threshold) led to an overestimate of the number of species in our sample, resulting in 56 OTUs. This result highlights a fundamental limitation of hierarchical clustering strategies for 16S rRNA analysis - only 42 of the 49 species present in our sample corresponded to a homogeneous sub-tree within the best hierarchical clustering of the data. The remaining 7 species cannot be correctly clustered irrespective of the similarity threshold chosen.

**Figure 2:**
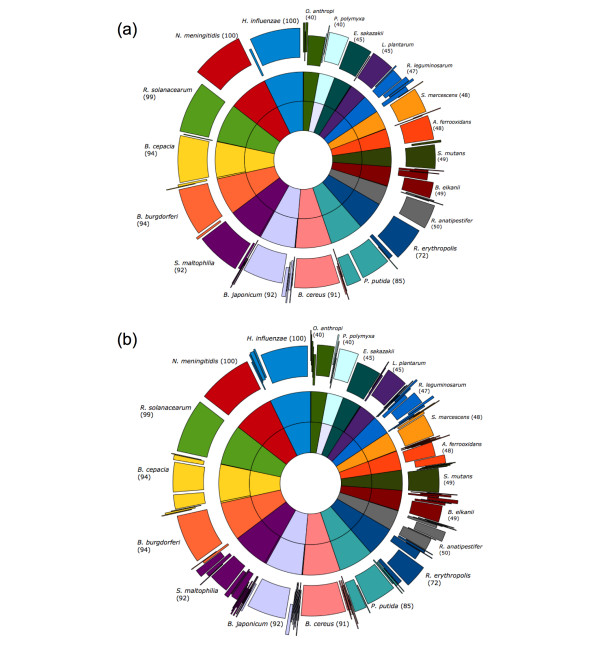
**Comparison of OTU sets to true species clusters**. The innermost rings represents the 20 most abundant species in the sample. Each species shown has ≥ 40 sequences in the dataset (total observations shown next to name of each species). The middle ring displays OTUs of the methodology using the parameters that resulted in the closest approximation of the species structure. The outer ring is an OTU set generated from methodologies used to study microbial communities of (a) soil [11] and (b) termite hindguts [39]. Previously published methodologies partition most species into several OTUs, resulting in an over-fragmentation of the species-level structure of the environment.

The results presented above highlight a wide variation in the OTU structure as we explore the parameters of the analysis process. To determine whether such variation is also present in the methodologies used in practice, we compared three analysis methodologies that performed well in our combinatorial search to several methodologies reported in published literature. The results shown in Table 2 indicate that the published methodologies can overestimate the diversity of the simulated environment, sometimes by more than 3-fold (see the "Termite hindgut" methodology). The fragmentation of the resulting OTUs is particularly striking among the most abundant phylotypes (Figure 2), where sequences belonging to the same species are distributed among multiple OTUs.

**Table 2:** OTU sets closest to the annotated species clustering for each multiple sequence alignment.

	Correction	MSA	Clustering	Distance	OTUs	Ace	Chao1	Shannon	VI
	F84	ClustalW	fn	0.05	56	79	116	3.39	0.044
Optimal	F84	NAST	fn	0.06	56	78	176	3.39	0.054
	JC	MUSCLE	fn	0.06	54	69	132	3.37	0.068
*Drosophila *(host) [36]	JC	ClustalW	fn	0.03	70	109	162	3.49	0.087
Marine sponge [37]	F84	ClustalW	fn	0.03	70	109	162	3.49	0.087
Soil [11]	JC	NAST	fn	0.03	99	150	169	3.66	0.157
Deep sea biosphere [13,38]	JC	MUSCLE	fn	0.03	96	396	466	4.66	0.190
Termite hindgut [39]	JC	NAST	fn	0.01	185	360	351	4.11	0.320

The "VI" column indicates the VI distance of each clustering from the annotated species clustering. Best methods chosen by our validation methodology for each MSA ("Optimal") are contrasted with five recently published methodologies. The "Correction" column corresponds to the evolutionary distance correction. Note that for the optimal methods using ClustalW and NAST alignments, the F84 and K2P corrections produced identical OTU sets because the distance matrices were very similar, though not identical. All methods in this table used furthest neighbor (fn) clustering. The Ace, Chao1, and Shannon diversity estimators are also provided.

### Nonparametric estimators of richness and diversity

**Figure 3:**
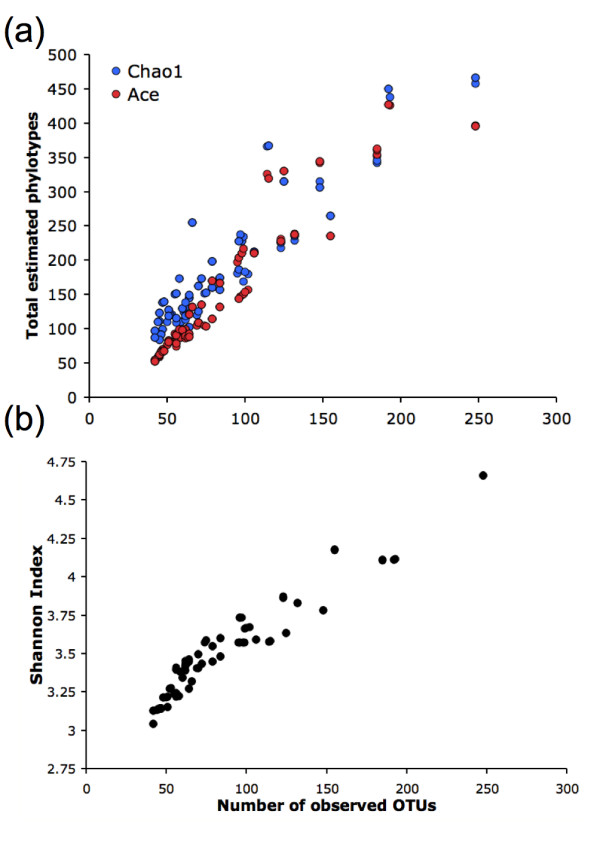
**Variability in nonparametric estimators and diversity indices using a clustering distances 0.01-0.05**. Plots of **(a) **Ace and Chao1, and **(b) **Shannon measures reveal significant sensitivity to OTU sets. Each plotted methodology used either the MUSCLE, ClustalW, or NAST MSA; they also used either furthest, nearest, or average neighbor clustering, and one of the following evolutionary distance corrections: JC, K2P, or F84.

The large variability in the OTU estimates produced by different methodologies also had a significant effect on commonly inferred ecological parameters. The Chao1 (*S*_*Chao*1_) [27] and ACE (*S*_*Ace*_) [28] richness estimators and the Shannon diversity (*H*) index [29] are measures commonly used to approximate the level of diversity present in an environment. Restricting our analysis to methodologies with distance thresholds from 0.01-0.05, these three measures were found to be highly sensitive to differences in OTU structure. Under the true (annotated) species clustering, *S*_*Ace *_= 57, *S*_*Chao*1 _= 67, and *H *= 3.41. *S*_*Ace *_and *S*_*Chao*1 _estimates for the computed OTU clusterings ranged from 52 to 427 and 84 to 466 phylotypes, respectively, while Shannon diversity indices ranged from 3.04 to 4.66 (Figure 3). Accurate estimates of the diversity of an environment are essential when planning whole-genome shotgun metagenomics sequencing projects, and an environment whose diversity is incorrectly perceived to be too high might not be studied at a metagenomic level.

### Partial masking of MSAs

**Figure 4:**
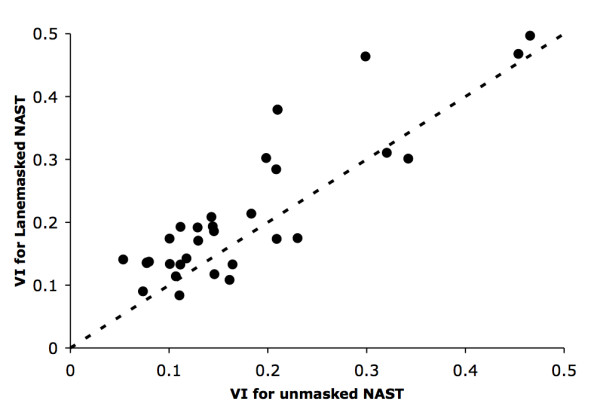
**Sensitivity of OTU structure to masking**. Comparison of OTU sets constructed with (x-axis) and without (y-axis) the application of a LaneMask to the multiple alignment generated by NAST. Both axes are labeled by the corresponding VI distance from the true species clustering. Overall, masked alignments resulted in poorer concordance to the true data labels. The dashed line is the identity function y = x.

To improve phylogenetic analyses, researchers often remove hypervariable segments of MSAs either manually or using a filter such as LaneMask [30,31]. We explored the impact of this approach on OTU clustering. Specifically, we used the GreenGenes LaneMask filter, which reduces a NAST alignment to 1287 highly conserved columns. The results are surprising - on average, employing LaneMask resulted in a worse approximation of the true species composition than the unmasked alignment (see Figure 4). This suggests that the use of a generic mask might be inappropriate when analyzing highly similar sequences with the goal of constructing OTUs, though its use may still be relevant to phylogenetic analyses of divergent sequences.

### Semi-supervised clustering alternatives

Our study has so far made the assumption that one of the primary goals of a 16S analysis pipeline is to estimate the composition of an environment at a pre-specified taxonomic level (e.g. species). As demonstrated by our results, the OTU methodologies proposed in the literature fail to achieve this goal, generally overestimating the number of species. Even by systematically evaluating various settings for the parameters of the analysis process, we could not obtain perfect concordance between the OTU structure and the species composition of the environment. This is in part due to the fact that the concept of "species" is born out of gross morphological and phenotypic traits of microorganisms, and therefore cannot be precisely mapped to fine-scale molecular measurements. Furthermore, the rate of evolution varies across the tree of life, making it unrealistic to rely on a single distance threshold.

As an alternative, we investigated the use of a semi-supervised clustering method to adaptively select a set of local distance thresholds that lead to OTUs that better fit the species composition of the environment. Specifically, we employed *VI-cut *[26], a clustering approach that identifies a cut within a hierarchical clustering tree that maximizes the fit with a labeled subset of the sequences. In the case of 16S analysis, VI-cut constructs a set of OTUs that optimally matches (in terms of VI distance) the species structure of an environment as inferred from a small subset of sequences that have known taxonomic assignments (for more details see Methods).

**Figure 5:**
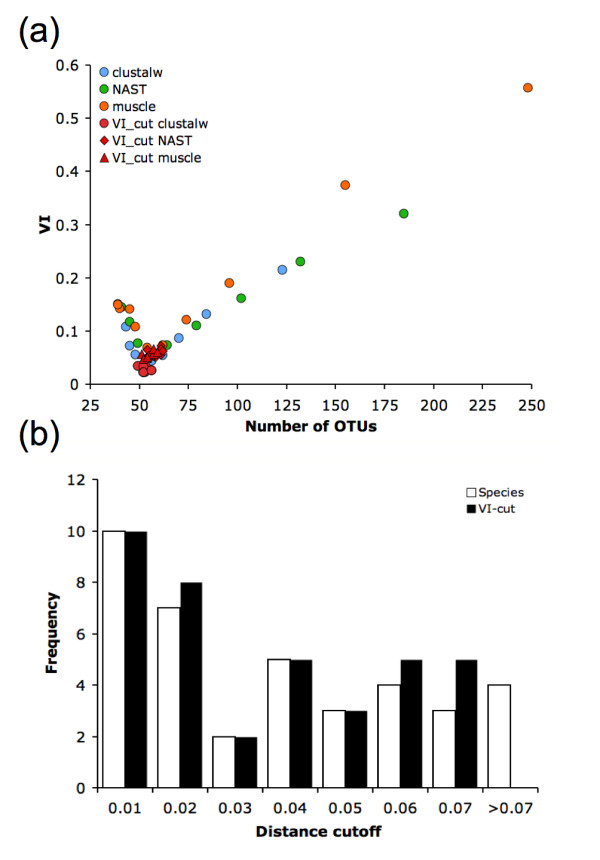
**Results of VI-cut compared to standard methodologies**. (a) Standard methodologies using a specific MSA with furthest neighbor clustering to find OTUs. VI-cut was employed using the same MSA and distance correction in each plot. For each VI-cut trial, 10% of the sequences were randomly selected and given labels. Over 20 trials, OTUs determined by VI-cut are stable and more accurate than the standard methodologies. (b) Distribution of distances within clusters defined by the species-annotation and a sample VI-cut clustering. Clusters with one member (*i.e*. singletons) are not shown. There is considerable variation (*D *= 0.01-0.07) in the optimal distance threshold among species.

We applied VI-cut to our data by simulating partial taxonomic knowledge of the dataset. For each MSA and the optimal distance correction (shown in Table 2), we randomly selected 10% of the sequences and provided VI-cut with their true labels. To assess the variability in the algorithm's results, we repeated this procedure 20 times. As seen in Figure 5A, VI-cut outperforms methodologies that employ a single distance threshold, irrespective of the MSA employed or the random selection of labeled sequences. The need for an adaptive threshold (such as that provided by the VI-cut approach) is highlighted in Figure 5B - the diameter of clusters corresponding to a single species in our data varies considerably among our sequences (from 0.01 to 0.07) and the semi-supervised learning algorithm implemented in VI-cut is able to closely approximate the true distribution of distance thresholds. Note that perfect concordance between OTUs and species cannot be achieved even with the best hierarchical clustering tree constructed from our data. It is an open question whether other clustering approaches could perform better in this context.

### Consistency of methods across multiple datasets

To investigate the consistent improvement of the VI-cut methodology over other methods, we created ten additional 16S environmental samples - each sample containing 500 randomly selected sequences from the original dataset. We repeated our comparison of VI-cut to other methods for these ten simulated samples. Examining the results across each MSA, we found that VI-cut consistently produced the best species-level approximation compared to standard methodologies (Table 3). Importantly, we point out that no MSA program was consistently optimal across all datasets.

**Table 3:** Top standard methodologies and performance of VI-cut.

MSA	Clustering	Distance	mean VI
MUSCLE	VI-cut	adaptive	0.0589
ClustalW	VI-cut	adaptive	0.0595
ClustalW	fn	0.03	0.0688
MUSCLE	fn	0.04	0.0691
ClustalW	fn	0.04	0.0697
MUSCLE	fn	0.05	0.0748
NAST	VI-cut	adaptive	0.0762
ClustalW	fn	0.02	0.0838
NAST	fn	0.05	0.0845
MUSCLE	fn	0.03	0.0860
NAST	fn	0.06	0.0872
ClustalW	fn	0.05	0.0942
NAST	fn	0.04	0.0992
MUSCLE	fn	0.06	0.1025
ClustalW	fn	0.06	0.1176
NAST	fn	0.03	0.1222
MUSCLE	fn	0.02	0.1370
ClustalW	fn	0.01	0.1505
NAST	fn	0.02	0.1633
NAST	fn	0.01	0.2362
MUSCLE	fn	0.01	0.2629

Methods are ranked by their mean VI-distance over 10 simulated datasets. We constrained the results to commonly accepted methods using furthest neighbor clustering and distance thresholds less than 0.07. A distance threshold of 0.01 is consistently among the worst performing methodologies. VI-cut consistently results in the best clustering for each MSA.

## Discussion and Conclusions

In this study we have shown how small differences in OTU methodologies can lead to significant variability in the resulting OTU structure, thereby affecting estimates of microbial diversity and ecological conclusions. Our results indicate that the most important factor in an OTU methodology is the distance threshold imposed during clustering, and that small changes in this parameter lead to a substantial variance in the estimated diversity of a community, making it difficult or even impossible to directly compare the results of studies utilizing different thresholds. Commonly employed thresholds of 0.01-0.03 (i.e. 97-99% similarity) fail to capture the underlying species composition of an environment and are frequently too stringent, producing inflated estimates of diversity. This result is in large part due to the fact that the overly-general biological definition of a species cannot be directly mapped at the fine-scale resolution provided by molecular-level observations. In addition, diversity estimates are highly sensitive to the abundance of rare members of a community and, thus, can be easily confused by the "noise" caused by sequencing errors, transient organisms, or "naked" DNA not originating from one of the members of the community. For all of these reasons, aggregate diversity measures do not necessarily correlate with the biological functions performed by members of a community, and must be augmented by additional, more specific, measurements of the community structure (e.g. focused on the most abundant members, or on individual sub-classes of organisms).

While the community simulated in our study was dominated by Proteobacteria, a significant proportion of the data belongs to other microbial phyla, thus the general results of our study likely hold in most other datasets. Furthermore, the organisms present in our samples are sufficiently distant at the 16S rRNA level to allow unambiguous clustering once a distance threshold was selected, i.e. the results we obtained reflect actual characteristics of 16S rRNA data rather than artifacts of the analysis process.

Our simulated samples were composed of a small number of high-quality sequences with exceptional length (>800 bp) - a relatively simple challenge compared to current datasets generated by pyrosequencing (Roche/454). 16S surveys employing pyrosequencing technologies generate considerably larger datasets (millions of sequences per sample), comprising reads of shorter lengths (~100-400 bp). The results we describe for long reads will only be exacerbated in the context of short-read data. Further, these data present several new computational challenges for OTU clustering including the need for faster alignment algorithms, the computation and storage of evolutionary distances (the size of distance matrices is proportional to the square of the number of sequences being analyzed), and the selection of distance thresholds appropriate for sequences with significantly less phylogenetic information than the Sanger-based reads used in our study. The analysis of the new sequencing data require the development of robust methods for OTU clustering, as well as rigorous validation of analysis methods. We finally demonstrated that a semi-supervised clustering approach (VI-cut) can significantly improve analysis quality, highlighting the potential for semi-supervised clustering approaches to fill the gap between the two extreme classes of approaches commonly used in analyzing 16S rRNA datasets: fully-supervised database searches that rely on expensive highly-curated datasets; and fully unsupervised OTU clustering procedures that use *ad hoc *similarity thresholds in an attempt to match poorly-defined taxonomic labels. Semi-supervised procedures such as VI-cut can generate accurate clusterings while only requiring high-quality labels for only a small subset of the sequences.

Finally, it is important to observe that clustering 16S rRNA sequences into a set of OTUs is a valuable analysis tool even if the resulting OTUs do not correlate with pre-defined taxonomic entities. However, the *ad hoc *choice of analysis parameters, in particular the selection of different distance thresholds, complicates cross-study comparisons and even basic descriptions of diversity. As 16S rRNA surveys are increasingly applied in a clinical setting [5,32-35] (e.g. to determine how microbiota correlate with human disease states), it is critical to accurately measure taxonomic diversity and identify individual species. Our results highlight the need for standardizing 16S rRNA analysis methods, or in the very least, reporting results obtained with multiple distance thresholds or clustering algorithms.

The data used in this study have been deposited in the FAMeS online database http://fames.jgi-psf.org - a repository for metagenomic analysis benchmarks [14]. Software used for semi-supervised clustering analysis is also available at http://www.cbcb.umd.edu/VICut/.

## Abbreviations

OTU: operational taxonomic unit; rRNA: ribosomal ribonucleic acid; VI: Variation of Information; MSA: multiple sequence alignment; RDP: Ribosomal Database Project; ANOVA: analysis of variance;

## Authors' contributions

JRW and SN ran the experiments and analyzed the data. M-RG provided computational expertise. JW, SN, NN, M-RG, CK and MP wrote the manuscript. All authors read and approved the final manuscript.

## Supplementary Material

Additional file 1 **Variation of information distances of high-level taxonomic clusterings from the annotated species clustering**. To give the reader some intuition about the VI distance metric, we computed VI distances between the annotated species-level clustering and other clusterings based on phylum, class, order, family, and genus annotations. This file contains a table of these reference distances. Click here for file
