# Supplementary material for: Alignment and clustering of phylogenetic markers - implications for microbial diversity studies
Source: BMC Bioinformatics. 2010 Mar 24;11:152. doi: 10.1186/1471-2105-11-152 (PMC2859756; doi:10.1186/1471-2105-11-152)
Supplement: Additional file 1 — Variation of information distances of high-level taxonomic clusterings from the annotated species clustering. To give the reader some intuition about the VI distance metric, we computed VI distances between the annotated species-level clustering and other clusterings based on phylum, class, order, family, and genus annotations. This file contains a table of these reference distances. [file 1471-2105-11-152-S1.DOC]

**Additional File 1.**

**Supplementary Table 1 - Variation of information (VI) distances of high-level taxonomic clusterings from the annotated species clustering.**

| Annotated clustering | VI |
| --- | --- |
| Phyla | 0.171 |
| Classes | 0.109 |
| Orders | 0.058 |
| Families | 0.026 |
| Genera | 0.008 |
| Species | 0 |

To give a set of reference VI-distances for known clusterings, we computed VI distances between clusterings based on annotated high-level taxonomic groupings (e.g. phyla or classes) and the corresponding species clustering. We see that clustering of sequences according to annotated genus is quite similar to the species clustering, and the VI distance increases as we move to higher taxonomic levels. Comparing the species clustering to itself results in a VI distance of 0.
